# Down memory lane: Unprecedented strong public and scientific interest in the “Spanish flu” 1918/1919 during the COVID‐19 pandemic

**DOI:** 10.1111/irv.12806

**Published:** 2020-09-18

**Authors:** Kaspar Staub, Joël Floris

**Affiliations:** ^1^ Institute of Evolutionary Medicine University of Zurich Zurich Switzerland; ^2^ Zurich Center for Integrative Human Physiology (ZIHP) University of Zurich Zurich Switzerland; ^3^ Institute of History University of Bern Bern Switzerland; ^4^ Digital Society Initiative (DSI) University of Zurich Zurich Switzerland; ^5^ Department of History University of Zurich Zurich Switzerland

1

To the Editor

Humans tend to place present events in the context of past experiences, especially during crises, when society's vulnerabilities become apparent. In these times, reflection on the learning processes from the past is initiated. All non‐pharmaceutical public health countermeasures currently being taken against the COVID‐19 outbreak are based on experience gained from past pandemics over the last several centuries,^1^, ^2^ which were mostly caused by influenza (1889/90, 1918/19, 1957‐59, 1968‐70, 1977/78, and 2009) and twice by coronaviruses (2003 and 2019/20). Among these pandemics, the 1918/1919 influenza outbreak (*Spanish flu*) remains the most devastating, as it caused an estimated 20‐100 million deaths worldwide and continues to exemplify the worst‐case scenario.^3^

**FIGURE 1:**
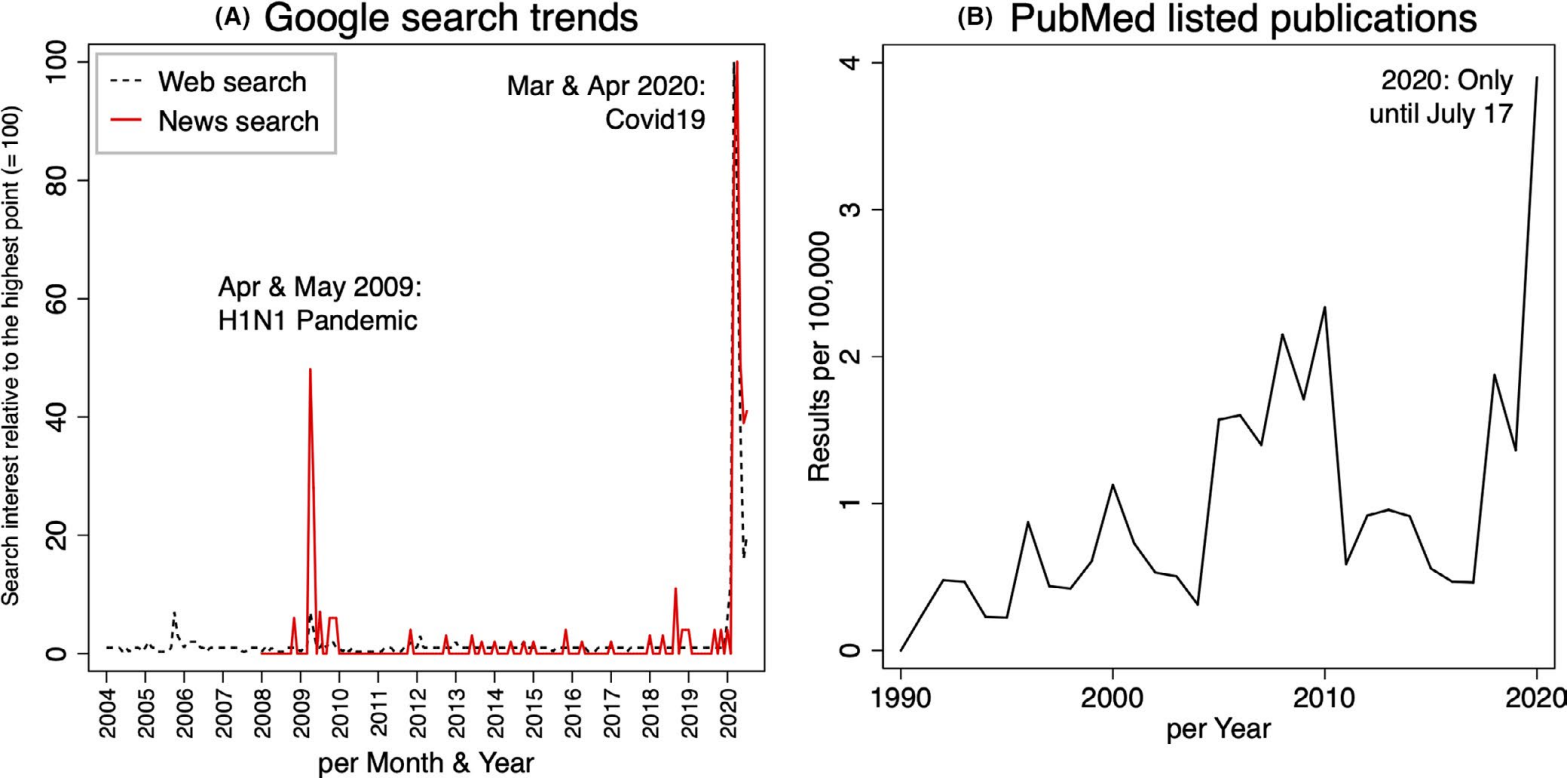
A, Results from the worldwide Google trends search per month and year using the term *Spanish flu* (black dashed line = web search; red line = news search). B, Yearly number of PubMed listed publications with the terms *Spanish flu* or *influenza 1918* in the title/abstract as a proportion of 100,000 listed items per year. (Date of searches: 17 July 2020)

Over the last few months, during the current worldwide COVID‐19 outbreak, we have noted a unprecedented and sharp increase in the public and scientific interest toward the influenza outbreak of 1918/1919. A first look at the Google search trends since 2004 (https://trends.google.com) revealed marked increases in searches for *Spanish flu* on the web and on news pages in March and April 2020 (Figure 1A). Further, according to a non‐systematic PubMed search using the tool *PubMed by Year* (https://esperr.github.io/pubmed‐by‐year/), the number of listed scientific studies per 100,000 citations using the search terms *Spanish flu* or *influenza 1918* in their abstract/title reached an all‐time high in 2020 (Figure 1B). Of the 31 studies that were published in 2020, some can still be attributed to the centenary of the Spanish flu. However, the number of studies explicitly referring to the Spanish flu in the context of COVID‐19 is increasing. Many of these publications tried to identify similarities and differences between the two pandemics in attempts to adapt the lessons of the past to current challenges.^4^

This pleasingly increased interest in the past and the lessons learned from in itself is not surprising. To highlight this fact however is important, because this valuable historical knowledge should be considered with thoughtfulness, especially since many questions about the Spanish flu are still unanswered.^5^ While research will certainly have more urgent problems to solve at the moment, in the future, historical epidemiologists should also analyze how researchers and the public recall the past during a new pandemic outbreak. What aspects of the past outbreaks are discussed where, in which context, and over which channels? Are these reflections scientifically balanced, and do they have an influence on the resilience and the reception and management of the current outbreak?

### AUTHOR CONTRIBUTION

**Kaspar Staub:** Conceptualization (equal); Formal analysis (equal); Investigation (equal); Methodology (equal); Supervision (lead); Visualization (equal); Writing‐original draft (equal); Writing‐review & editing (equal). **Joël Floris:** Conceptualization (equal); Formal analysis (equal); Investigation (equal); Methodology (equal); Visualization (equal); Writing‐original draft (equal); Writing‐review & editing (equal).

### Peer Review

The peer review history for this article is available at https://publons.com/publon/10.1111/irv.12806.

REFERENCES1

Giles‐Vernick
T
, 
Craddock
S
, 
Gunn
J
. Influenza and Public Health. Learning from Past Pandemics. London: Earthscan from Routledge; 2015:293.2

Webb
JLA
. The historical epidemiology of global disease challenges. Lancet. 2015;385:322‐323.2571383310.1016/s0140-6736(15)60108-83

Murray
CJ
, 
Lopez
AD
, 
Chin
B
, 
Feehan
D
, 
Hill
KH
. Estimation of potential global pandemic influenza mortality on the basis of vital registry data from the 1918–20 pandemic: a quantitative analysis. Lancet. 2006;368:2211‐2218.1718903210.1016/S0140-6736(06)69895-44

Morens
DM
, 
Daszak
P
, 
Taubenberger
JK
. Escaping Pandora’s Box — Another Novel Coronavirus. N Engl J Med. 2020;382:1293‐1295.3210166010.1056/NEJMp20021065

Taubenberger
JK
, 
Kash
JC
, 
Morens
DM
. The 1918 influenza pandemic: 100 years of questions answered and unanswered. Sci Transl Med. 2019;11(502):eaau5485.3134106210.1126/scitranslmed.aau5485PMC11000447
